# Possible prophylactic effect of omega-3 fatty acids on cadmium-induced neurotoxicity in rats’ brains

**DOI:** 10.1007/s11356-019-06259-8

**Published:** 2019-08-29

**Authors:** Hanan S. Alnahdi, Iman A. Sharaf

**Affiliations:** 1 Department of Biochemistry, Faculty of Science –Alfaisaliah, King Abdulaziz University, PO Box 50212, Jeddah, 21523 Saudi Arabia; 2 Department of Biochemistry, Faculty of Science –Alfaisaliah, University of Jeddah, PO Box 50212, Jeddah, 21523 Saudi Arabia

**Keywords:** Cadmium, Neurotoxicity, Omega-3 fatty acid, Prophylactic effect, Neurodegenerative disorders, Biochemical changes

## Abstract

Cadmium (Cd) has long been noted to induce neurodegenerative disorders. Therefore, this study aimed to assess the toxicological impact of Cd on rat brains and evaluate the possible ameliorative impact of omega-3 fatty acids as a protective agent of nervous system. Rats were divided into four groups: group I supplemented orally with saline; group II intoxicated with CdCl_2_ (5 mg/kg b.w. orally), and groups III and VI supplemented with omega-3 (100 mg/kg b.w. orally) simultaneously or before CdCl_2_ administration, respectively. Cd intoxication induced biochemical and histopathological disturbances in treated rats. Omega-3 fatty acid considerably improved the Cd-associated biochemical changes, reduced the elevation of lipid peroxidation, and normalized the Cd impact on the levels of superoxide dismutase, catalase, glutathione-S-transferases, 8-hydroxydeoxyguanosine, heatshock protein70, nuclear factor-κB, and interferon-γ as well as of neuronal enzymes such as acetylecholinesterase and monoamine oxidase within the brains of treated rats. Additionally, histological findings supported the results that Cd treatment-induced neurodegenerative changes and that polyunsaturated fatty acids act as antioxidants and neuroprotective agents against Cd toxicity. Co-treatment with omega-3 fatty acid was more beneficial than pretreatment. Thus, omega-3 fatty acid should be included in diet to prevent or suppress neurodegenerative disorders caused by continuous exposure to Cd.

## Introduction

Cadmium (Cd) is a certainly occurring heavy metal possessing extreme dangers to human health. It is far recognized to be one of the most toxic environmental and business pollutants that cause water, air, and food pollution (Tchounwou et al. 2012). Cd is specific among different metals due to its toxicity at a totally low dosage, long biologic half time of life, and its low rate of excretion from the body. Commercially, it is used in TV screens, lasers, batteries, paint pigments, cosmetics, galvanizing metallic, copper alloys, rubber and plastics stabilizers, fungicides, and in lots of different products (Kaoud and Mekawy 2011). Green food is also an important supply of access of Cd and populations, which includes farmers that eat domestically grown products that are at unique threat (Mukherjee et al. 2010). Cd has been observed also in liquids, fish, meat, milk, eggs, and cereals; and also present in cigarettes (Afifi and Embaby 2016). Blood–brain barrier (BBB) restricts the entry of foreign molecules, but in the occasion of continual minimum exposure of Cd, it disrupts the BBB integrity and adversely impacts the brain (Mizee and De Vries 2013). Post-BBB entry of Cd provokes neurotoxicity with an array of clinical signs such as behavioral changes, degeneration of neurons, and alteration of nerve transmitters (Renugadevi and Miltonprabu 2009). Similarly, it has been shown that kids exposed to Cd may additionally develop studying disabilities and hyperactivity disorders (Wang and Du 2013). Short-term exposure of Cd might prelude to Parkinson’s disease (Caudle et al. 2012).

Brain consists of high concentration of lipids that are liable to oxidative radical attack throughout the Cd exposure. There exists a rampant generation of radicals precisely, superoxide radical that overture to induce lipid peroxidation (LPO), membrane macromolecular damage, altered antioxidant system, altered gene expression, and programed cell death (Ma et al. 2017). As oxidative stress is one of the necessary mechanisms of cadmium-induced damages, it will be expected that the administration of some antioxidants ought to be a crucial therapeutic approach (Renugadevi and Prabu 2010). A growing area of study is examining the neurobehavioral elements of omega-3 unsaturated fat (alpha-linolenic, docosahexanoic [DHA], and eicosapentanoic acids) and the simple part of these fundamental fats inside the central nervous system. DHA is considerable within the brain (Kim et al. 2014) and had a capacity to affect signaling pathway (Crawford 2013). Incorporation of DHA into cell membranes affects in lowering lipid peroxidation and oxidative pressure in neurons. The DHA also reduces proinflammatory mediators and anti-inflammatory compounds in conjunction with materials that defend brain cells known as neuroprotectins (Bazan et al. 2011). There are no reviews regarding the usage of omega-3 fat as a prophylactic agent against the toxicological effect of cadmium exposure in nervous system as it is far widely used as antioxidant and anti-inflammatory. Consequently, the intention of the prevailing examine was to evaluate the toxicological impact of Cd on rat brain. The study also might be extended to explore the effect of omega-3 fatty acid as a protecting agent with antioxidants and anti-inflammatory residences in a trial to minimize or suppress the cytotoxic impact of metallic Cd on nervous system. Oxidative stress, inflammatory, DNA injury markers, and neuronal enzymes were determined. Histopathological studies of rat brain tissue might be examined to affirm the biochemical investigations.

## Materials and methods

### Chemicals

Cadmium chloride was bought from Merck, Darmstadt, Germany, and dissolved in distilled water before administration. Omega-3 fatty acids were obtained from Abbott product GmbH, Germany. Other chemicals and reagents used were of analytical grade.

### Experimental layout

This study comprised 60 male albino rats (150–200 g body weight) divided into four groups, 15 rats each. The animals were housed in cages. The utilization of animals and experimental design were approved by unit of biomedical ethics, King Abdulaziz University Medical faculty, Jeddah, KSA, which are in compliance with the national and international laws and policies (7th edition). All procedures were performed per the National Institutes of Health Guiding Principles within the Care and Use of Animals. Animals were allowed to acclimate at the experimental surroundings for 2 days before dosing initiation. The animals were divided into four groups (*n* = 15 each). Group I served as management. Group II was supplemented with cadmium chloride in a dose of 5 mg/kg b.w. orally for 6 days dissolved in water (Kaoud and Mekawy 2011). Group III was supplemented as in group II accompanied by oral supplementation of omega-3 fatty acid with a daily dose of 100 mg/kg weight for a period of 6 days (El-Ansary et al. 2011). Group IV was supplemented orally with omega-3 fatty acid with associate daily dose of 100 mg/kg weight for period of 6 days, followed by supplementation with Cd as in group II. Rats were scarified by decapitation when night-long fast (12–14 h), and also the brains were removed. Parts of the brains had been preserved in 10% neutral buffered formalin solution for histopathological study.

### Biochemical investigations in the brain tissues

Oxidative stress parameters, malondialdehyde (MDA), superoxidedismutase (SOD), catalase (CAT), and glutathione-S-transferases (GST), in the brain tissue homogenate, were measured utilizing commercial kits (Nanjing, Jiancheng Co., China). Nervous system enzymes and acetyl choline esterase were estimated using commercial assay kits according to the manufacturer’s instructions, and monoamine oxidase was estimated using enzyme-linked immunosorbent assay (ELISA) kit (Life Span Bioscience, Inc.). DNA damage detected by estimation of 8-hydroxydeoxyguanosine (8-OHdG) and heatshock protein70 (Hsp70) which were measured in the brain homogenates using ELISA kits (Uscn Life Science Inc., Wuhan, China) in accordance with the instructions supplied by manufacturer. Nuclear factor-κB (NF-κB) and interferon-γ (IFN-γ) were measured as proinflammatory markers using an ELISA kit (a product of Thermo Scientific, Waltham, MA, USA) following the instructions of the manufacturer.

### Histopathological studies

Brain tissues were examined to evaluate the histomorphological changes in different experimental groups. Samples of brain tissues were collected and fixed in 10% formaldehyde for 24 h. Some sections of the samples were stained with hematoxylin and eosin for examination by ordinary optical microscope to evaluate the cytotoxic effects of Cd administration (Bancroft 2008).

### Statistical analysis

A computer SPSS application could be used, and the results had been expressed as suggested ± SD (*n* = 15). Comparisons might be made by way of the one-way ANOVA between the management and treated groups. Dunnett’s check might be used to compare between the corporations.

## Results

### Biochemical study

**Fig. 1:**
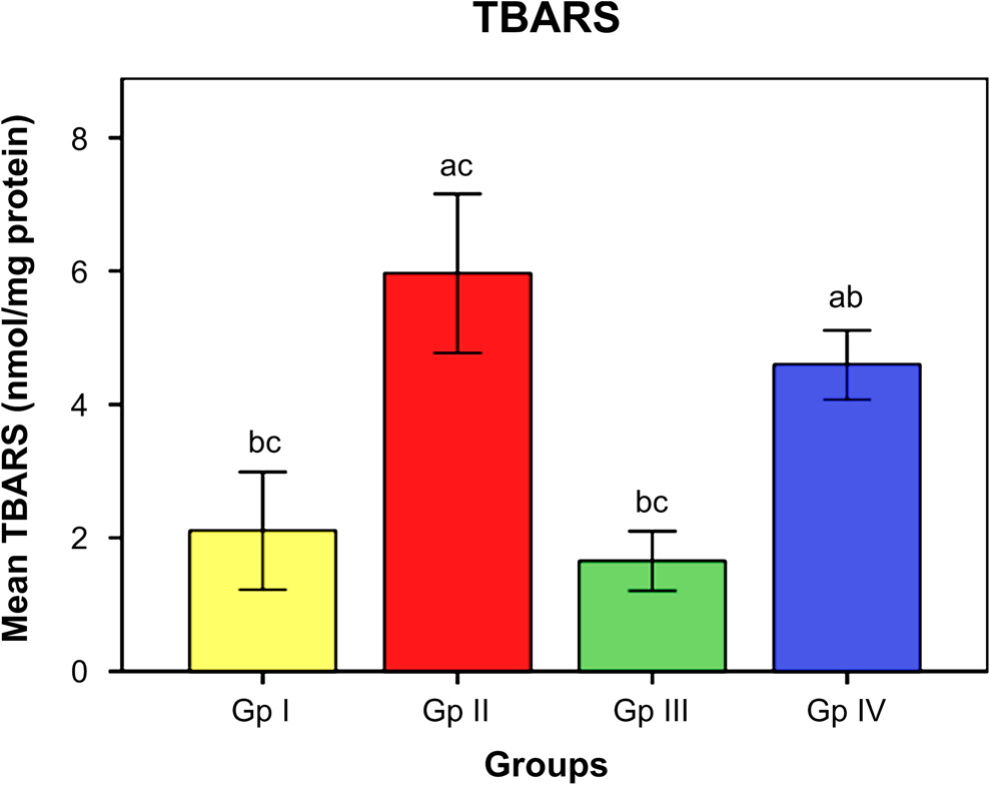
Effect of omega-3 on brain lipid peroxidation (TBARS) in rats intoxicated with Cd. GI control, GII Cd-intoxicated rats, GIII Cd-treated rats simultaneously with omega-3, GIV omega-3-treated rats before Cd intoxication. Data are presented as mean ± S.E. of 15 rats at *p* < 0.05. (a) Significant difference vs. control, (b) significant difference vs. GII, and (c) significant difference vs. GIV

**Fig. 2:**
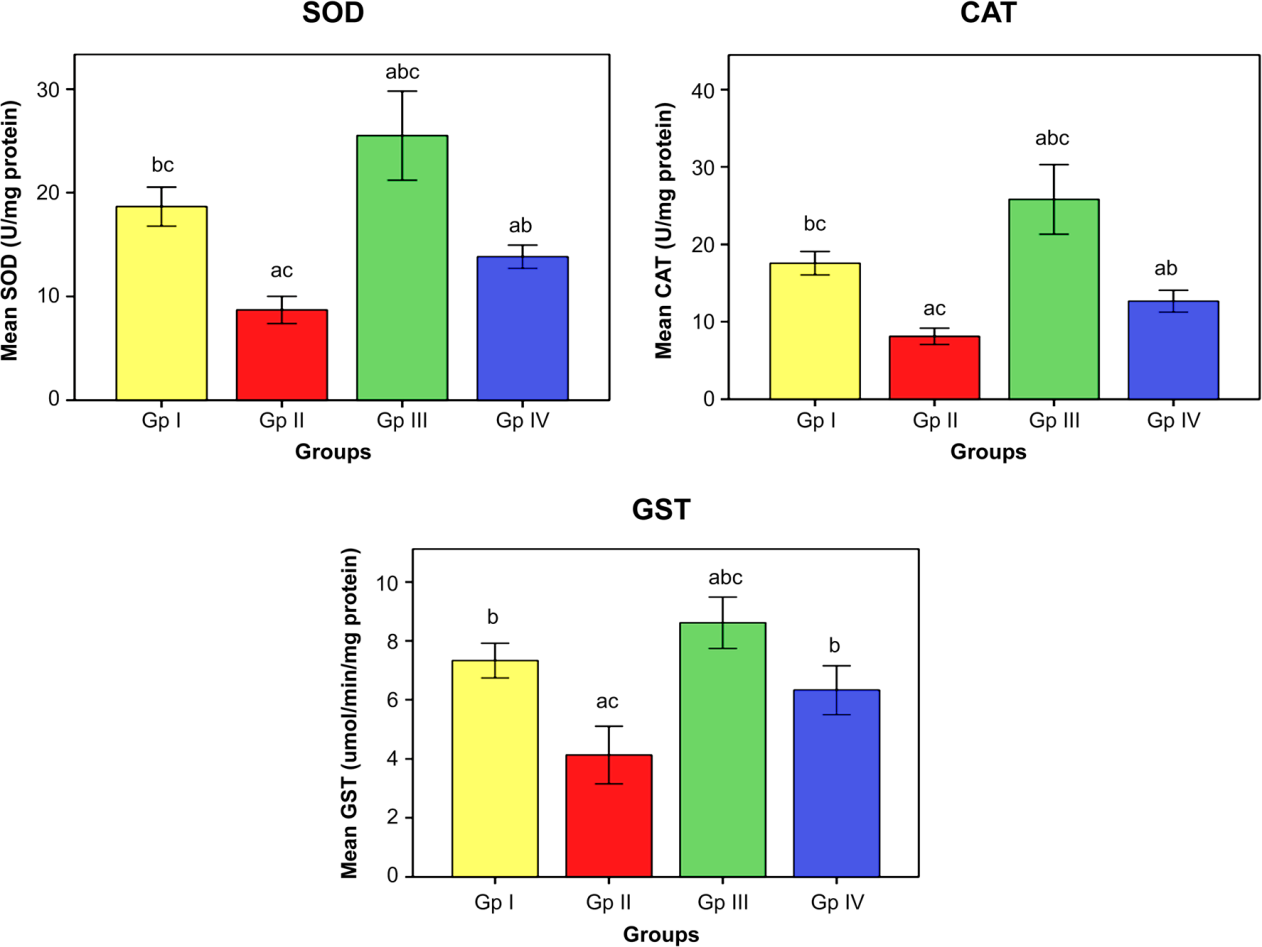
Effect of omega-3 on brain antioxidant markers (SOD, CAT, and GST) in rats intoxicated with Cd. GI control, GII Cd-intoxicated rats, GIII Cd-treated rats simultaneously with omega-3, GIV omega-3-treated rats before Cd intoxication. Data are presented as mean ± S.E. of 15 rats at *p* < 0.05. (a) Significant difference vs. control, (b) significant difference vs. GII, and (c) significant difference vs. GIV

**Fig. 3:**
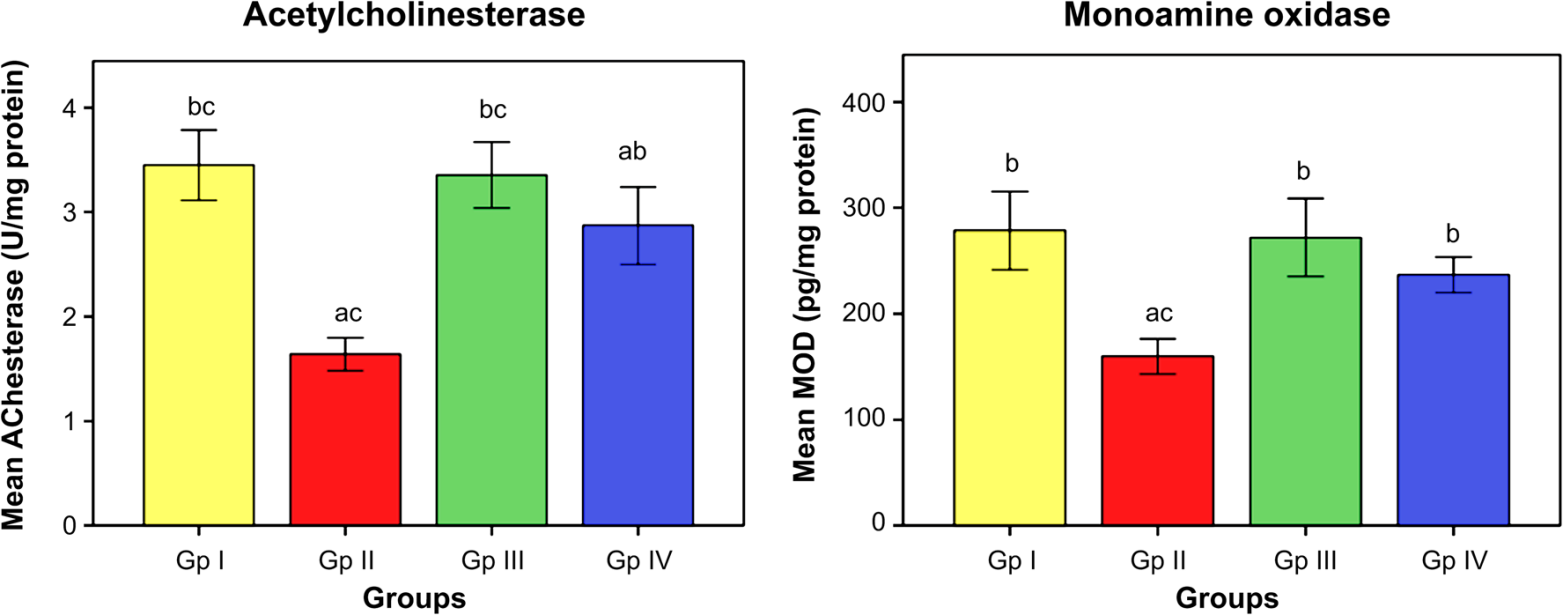
Effect of omega-3 on brain nervous system enzymes, acetyl cholinesterase (AChE), and monoamine oxidase (MAO) in rats intoxicated with Cd. GI control, GII Cd-intoxicated rats, GIII Cd-treated rats simultaneously with omega-3, GIV omega-3-treated rats before Cd intoxication. Data are presented as mean ± S.E. of 15 rats at *p* < 0.05. (a) Significant difference vs. control, (b) significant difference vs. GII, and (c) significant difference vs. GIV

**Fig. 4:**
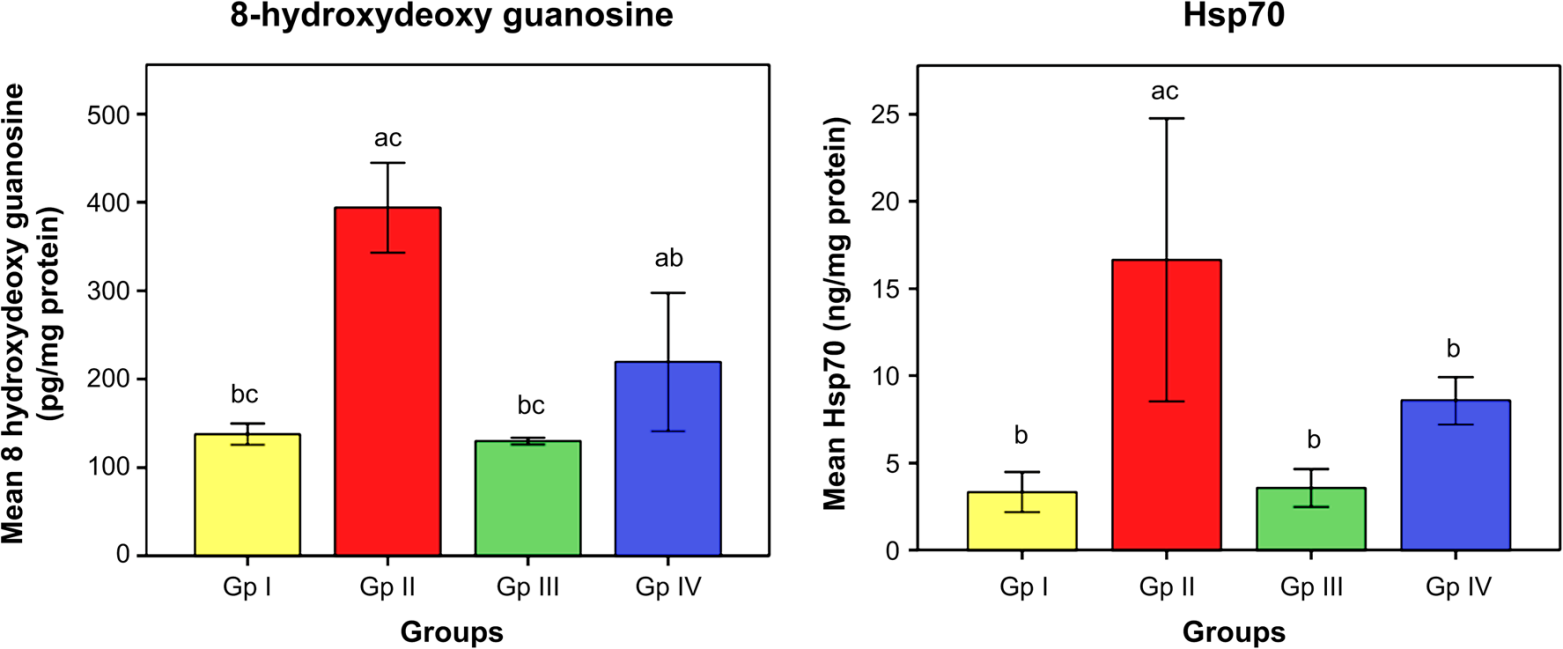
Effect of omega-3 on brain 8-hydroxydeoxyguanosine (8OHdG) and heat shock protein70 (Hsp70) in rats intoxicated with Cd. GI control, GII Cd-intoxicated rats, GIII Cd-treated rats simultaneously with omega-3, GIV omega-3-treated rats before Cd intoxication. Data are presented as mean ± S.E. of 15 rats at *p* < 0.05. (a) Significant difference vs. control, (b) significant difference vs. GII, and (c) significant difference vs. GIV

**Fig. 5:**
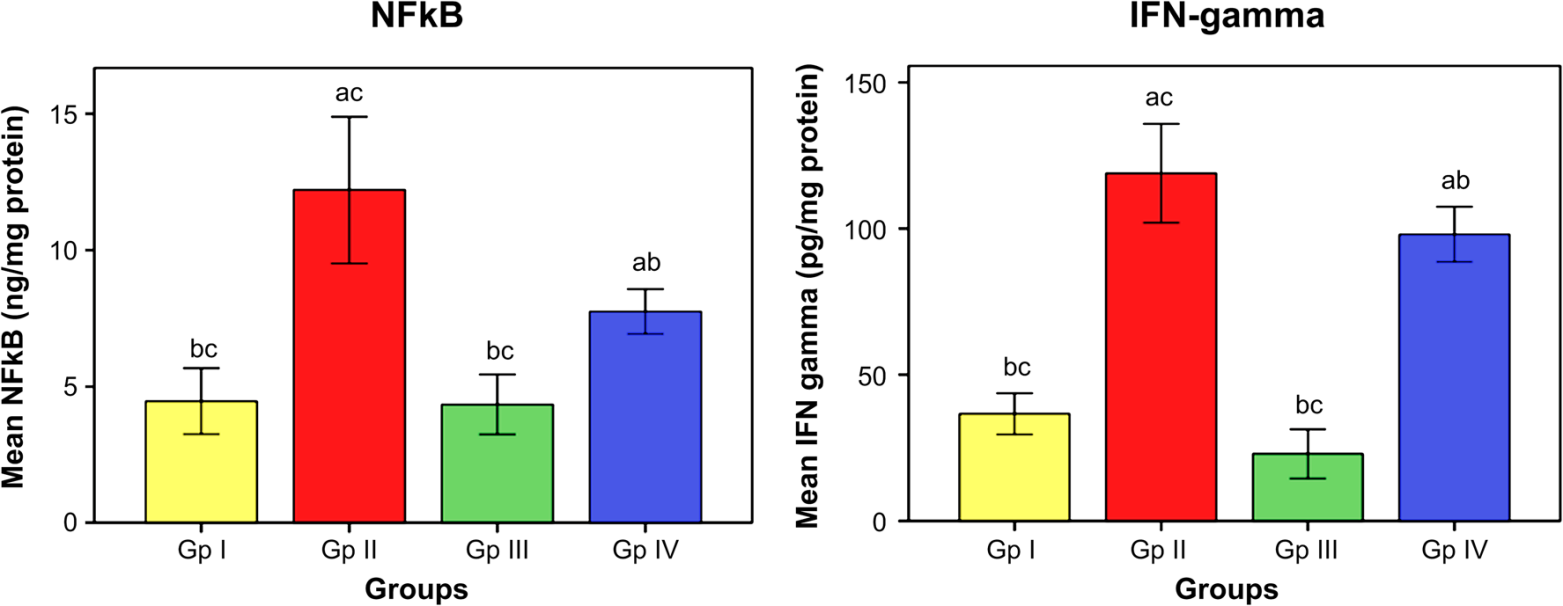
Effect of omega-3 on brain nuclear factor-κB (NF-κB) and interferon-γ (IFN-γ) in rats intoxicated with Cd. GI control, GII Cd-intoxicated rats, GIII Cd-treated rats simultaneously with omega-3, GIV omega-3-treated rats before Cd intoxication. Data are presented as mean ± S.E. of 15 rats at *p* < 0.05. (a) Significant difference vs. control, (b) significant difference vs. GII, and (c) significant difference vs. GIV

The influence of omega-3 fatty acids on the level of oxidative stress and antioxidant indices is demonstrated in Figs. 1 and 2. The data illustrated that subjection of rats to Cd toxicity (GII) pronouncedly boosted the level of brain MDA (index of membrane lipid oxidation) and diminished the brain antioxidant enzymes, namely SOD, CAT, and GST with respect to control animals (GI). Ingestion of omega-3 fatty acids simultaneously with CdCl_2_ (GIII) or before CdCl_2_ (GIV) administration, successfully ameliorating the brain levels of oxidative stress index (MDA) and the enzymes of antioxidants in Cd-intoxicated rats compared to intoxicated untreated ones. Data in Fig. 3 reveals the brain concentrations of nervous system enzyme indices [acetylcholinesterase (AChE) and monoamine oxidase (MAO)] in control and Cd-intoxicated groups. The results showed that Cd toxicity caused significant decrease in AChE and MAO versus control rat (*P* < 0.05). Supplementation of Cd-intoxicated rats with omega-3 fatty acids simultaneously with or before Cd intoxication markedly significantly increases (*P* < 0.05) the activities of enzymes and ameliorated it versus Cd-intoxicated animals. Figure 4 shows a significant elevation (*P* < 0.05) of the brain 8OHdG and HSP70, as indicators of DNA disfiguration, in Cd-intoxicated rats with relation to control ones. Treatment of Cd-intoxicated rats with omega-3 fatty acids simultaneously with or before Cd administration obviously modulated the increase in 8OHdG and HSP70 compared to BCD intoxicated rats. Figure 5 illustrates the efficacy of omega-3 fatty acids on the brain concentrations of inflammatory molecules, including IFN-γ and NF-κB, in Cd-intoxicated rats. The data demonstrated that Cd toxicity significantly (*P* < 0.05) stimulated the generation of these proteins in comparison to control animals. The intake of omega-3 fatty acids simultaneously with or before Cd administration significantly depleted the brain IFN-γ and NF-κB in Cd-intoxicated rats versus control ones.

### Light microscopic examination

**Fig. 6:**
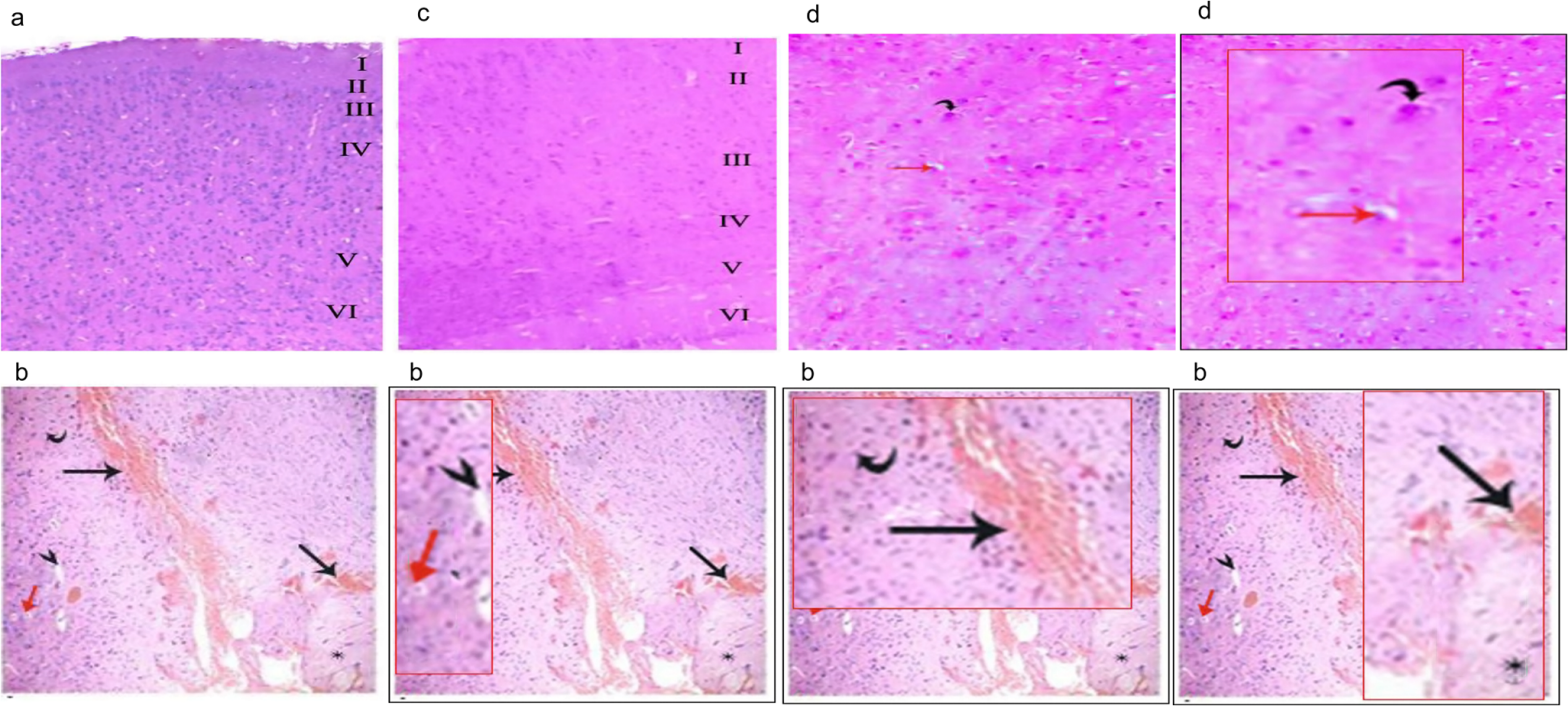
Histopathological examination of rat brains intoxicated with Cd. Paraffin sections stained with hematoxylin and eosin (× 200) are shown. **a** Section of control rat brains showing the typical layered appearance of the cerebral cortex labeled (I–VI) as follows: I, Molecular layer; II, External granular layer; III, Pyramidal cell layer; IV, Internal granular layer; V, Gaglionic layer; and VI, Multiformlayer. **b** Section of Cd-intoxicated rat brain showed neuronal cell disorganization and hypercellularity, dilated blood vessel (arrowhead), increased apoptotic cells (red arrow), areas of severe hemorrhage (black arrows), vacuolations of neuropil (*), and few red neurons (curved arrow). **c** Section of rat brain treated with omega-3 simultaneously with Cd showed normal histological structure of the brain. **d** Section of rat brain treated with omega-3 before Cd showed reduced degenerative changes

Stained sections of rats’ brain of the group I (control group) showed normal cerebral cortex with its six layers (molecular, external granular, pyramidal, internal granular, ganglionic, and the deepest multiform layer), as shown in Fig. 1. Brain tissues of group II (Cd treated group) showed degenerative changes. These include neuronal cell disorganization and hyper cellularity, dilated blood vessel, increased apoptotic cells, severe hemorrhage, vacuolations of neuropil, and few red neurons as shown in Fig. 2. No histopathological abnormalities were observed in group III (Cd^+^ omega-3 fatty acid-treated group). Brain tissues of group IV (omega-3 fatty acids followed by Cd intoxication) showed return of brain tissues toward normal morphology but still different when compared with control (Fig. 6). The biochemical and histological results showed that the ameliorating effect of co-administration of omega-3 with Cd was (beneficial) than protection with omega-3 before Cd intoxication.

## Discussion

Although Cd toxicity is currently documented in virtually each organ, the central nervous system remains extremely conscious of low levels of Cd exposure. Accumulating evidence indicates that Cd causes a rise in the production of ROS and neuronal cell death (Patra et al. 2011). The CNS is enriched with high amounts of the lipid, and so, it is at an additional risk of the attack of free radicals generated throughout oxidative neurotoxicity. The cardinal event within the Cd-evoked neurotoxicity is the ability to penetrate BBB and collocate within the brain to elicit lipid peroxidation. Cd-evoked lipid peroxidation is mediated by overproduction of superoxide radical to make a toxic product, MDA (El-Tarras et al. 2016). Further, Cd-evoked free radicals could cause the oxidation of macromolecules to make carbonyl groups, which is a toxicant method throughout Cd-mediated neurotoxicity (Engström et al. 2010). In this study, we determined an elevated level of MDA in brain tissues of rats intoxicated with Cd, while the levels of antioxidant enzymes SOD, CAT, and GST were diminished. Treatment with ω-3 induced a major reduction in elevated levels of MDA and elevation of antioxidant enzymes (Valavanidis et al. 2009). Similarly, previous investigation expressed that polyunsaturated fatty acids contained an effective potential in minimizing oxidation of lipids in experimental animal model (Gharami et al. 2015). Our results were confirmed by Ozen et al. (2008) who have shown that omega-3 supplementation after ischemic reperfusion reduced the level of MDA and increased the level of SOD. The brain antioxidant defensive system network encompasses a series antioxidant enzyme, SOD, CAT, and GST, and the primary response is elicited by SOD against the ROS and per oxidative stress. Meanwhile, the superoxide radical is initially generated in the ROS system and converted into H_2_O_2_ and molecular oxygen by CAT or GST. Thus, neuronal tissues are more susceptible to oxidative stress attack which may be attributed to the diminished antioxidant levels (Birben et al. 2012). The attainable mechanisms in Cd-elicited antioxidant enzyme depletion are also because of the interaction with SH groups in some enzymes to exchange essential metals from their active sites, or alteration in amino acid chain because of the radical mediate reaction. Finally, these toxic changes result in inactivation and loss of protein function (Sharma et al. 2014). SOD, presumably Cu/ZnSOD, known to be expressed in CGNs (Okabe et al. 2000) is incredibly sensitive and is inactivated by Cd treatment. It was postulated that Zn can be replaced by Cd in the catalytic site of the enzyme leading to the reduction in SOD activity (Casalino et al. 2002). The same mechanism has been recommended to be concerned within the interaction between Cd and the catalytic subunit of CAT resulting in the reduction of its activity (Cuypers et al. 2010). Thus, our findings presented data concerning the behavior of antioxidant enzymes against Cd toxicity in CNS. Administration of ω-3 with or before Cd intoxication refurbished the depleted antioxidant enzymes by inhibition of lipid peroxidation and oxidative stress (Avramovic et al. 2012). It is documented that DHA is consumed by the brain and is incorporated into the nerve cell membranes (Rapoport et al. 2011), which confirmed the neuroprotective effects of omega-3 against the Cd-induced oxidative stress. In this study, the activities of AChE and MAO in the brain were considerably attenuated in Cd-intoxicated rats that correspond with the results by Pari and Murugavel (2007). The study of brain enzyme activities such as AChE is crucial in detecting the toxic effects of certain heavy metals. Since brain AChE activity is a crucial controller of the behavioral action, the attenuated activity of AChE within the brain may be one among the symptoms for Cd-elicited complication in the brain (Pari and Murugavel 2007). Many studies elucidated that the radical production may be related to the decrease in the activity of AChE in the brain (Pervin et al. 2014). Similarly, metallic cadmium will go through the BBB and concentrated into the brain that is well at risk of Cd-induced peroxidation and oxidative stress. Reports counsel that Cd binds with AChE and alters the enzyme structure to provide unreactive species (Ma et al. 2017; Patra et al. 2011). MAO has the ability to limit the action of many necessary neurotransmitters, and oxidatively alter neurochemical and xenobiotic amines thereby fixing the idea of fast repetitive response. MAO plays a vital role in catabolizing the neuroactive amines. The reduced level of MAO ascertained within the present study suggests that Cd has the potential to achieve the CNS and impair its function. Meanwhile, treatment with omega-3 considerably enhanced the amount of MAO and AChE and come back to close the conventional levels. In agreement with the current findings, Ibrahim et al. (2018) found that the protection and daily treatment (for 7 days) of mouse model of Parkinson’s disease (PD) with omega-3 reversed the decrease in AChE and MAO activities toward a rise. This observation may represent the mechanism by which omega-3 fatty acids act to attenuate the increase within the cholinergic and serotonergic activity. These useful effects of omega-3 fatty acid may well be attributed partly to its antioxidant and anti-inflammatory potential. The formation of ORS with depletion in antioxidants could cause pathological injury at the molecular levels. The nucleotide pool is one of the targets of ORS attack, and guanine is specifically susceptible to chemical reaction because of its low oxidation–reduction potential; 8-hydroxy-2′-deoxyguanosine (8-OHdG) is the major form of the deoxyribonucleic acid adduct, and analysis of the concentration of this deoxyribonucleic acid adduct is employed as an associate indicator of oxidative deoxyribonucleic acid disfiguration (Ming et al. 2014). Parallel with a former report, this research illustrated that exposure of animals to Cd toxicity promoted brain deoxyribonucleic acid injury as tested by enhanced 8-OHdG (Engström et al. 2010). The correlation between Cd-generated ORS and deoxyribonucleic acid injury has been contributed to the assembly of 8-OHdG, a basic signal for ORS formation and tumor genesis (Valavanidis et al. 2009). Oxidative deoxyribonucleic acid injury caused by Cd exposure could attribute to the potential of Cd to displace the essential bivalent metals, zinc, requiring enzymes that maintain deoxyribonucleic acid integrity, leading to the inactivation of those enzymes. Deoxyribonucleic acid injury by Cd could promote cell cycle stop and eventual death (Engström et al. 2010). Our result could counsel that production of 8-OHdGs due to Cd toxicity could be an important risk factor in the development of brain malignancy. Supplementation of rats with omega-3 fatty acids at the same time with or before Cd toxicity considerably attenuated the brain increase in 8-OHdG compared with Cd-intoxicated rats. In line with our result, some authors reported that omega-3 fatty acid may modulate the increase in serum 8-OHdG in cigarette smokers (Ghorbanihaghjo et al. 2013). The current study suggests that the power of omega-3 fatty acids to counteract the oxidative alteration of DNA may be due to their antioxidant potential impacts. The present work showed a marked rise within the brain HSP70 of Cd-treated rats with regard to management ones. Elevation in this protein in the brain of Cd-intoxicated rats is considered another sign for brain stress (oxidative stress and inflammatory stress). This result is confirmed by Jing et al. (2013) who suggest the primary proof in the induction of HSP70 by metallic element. These proteins act as cyto protective, maintaining cell survival by preventing the misfolding or degradation of proteins in numerous stress conditions (Oka et al. 2013). The present investigation might recommend that the rise of brain HSP70 in Cd-treated rats is considered as a detoxification defense mechanism against Cd toxicity. Intake of omega-3 fatty acid at the same time with or before Cd administration markedly reduced the rise in HSP70 level versus Cd-untreated rats. This investigation provides that the first proves that omega-3 fatty acids will modulate the brain expression of HSP70 in rats underneath the cytotoxic impact of Cd. The modulating impact ofomega-3 fatty acid on brain HSP70 might assign to their ability to ameliorate the OS and inflammation in response to Cd toxicity.

Results of the present work determined that exposure of rats to Cd elicited inflammation in their brains as determined by over-expression of IFN-γ in Cd-treated rats compared with management animals. It is reportable that exposure to Cd will cause general inflammation because of the downstream impact of cadmium promoted OS (Souza et al. 2004). Under pathological conditions, IFNγ within the brain is increased because of brain injury or aging-associated enhanced porosity of the BBB. Moreover, the previous data demonstrated that IFNγ priming potentiates the assembly of ROS and NO by ATP-stimulated microglia (Spencer et al. 2016) and causes Ca^2+^ flow from the extracellular medium (Tao-Cheng 2002). The increase in intracellular Ca^2+^concentration stimulates activity of Ca^2+^/calmodulin-dependent protein kinase II, resulting in enhanced iNOS expression and subsequent NO production (Tao-Cheng 2002). NF-κB is a very important inflammatory inducible transcription issue. Activation of this issue regulates the expression of many inflammatory molecules. The present work depicted over-expression of brain NF-κB in Cd-treated rats versus management animals. It is rumored that mice exposed to Cd toxicity elicited over generation of NF-κB and inflammatory cytokines (Lee and Lim 2011). NF-κB activates several inflammatory genes, leading to tissue injury within the nervous system, and activation of NF-κB is incontestable in such pathological conditions as acute or chronic neurological disorders as PD (Breton et al. 2013; Armand and Darvakh 2015). Besides, NFκB has been found to possess a basic role in generation of NO via the generation of inducible NO synthase (Spitzer et al. 2002). Supplementation of polyunsaturated fatty acids to Cd-intoxicated animals pronouncedly diminished the rise of brain IFN γ and NFκB as compared to Cd-intoxicated rats. This result implies the potential anti-inflammatory and immunomodulatory functions of polyunsaturated fatty acid. The anti-inflammatory useful impact of polyunsaturated fatty acid has been confirmed (Calder 2010). Suppression of brain IFN-γ and NF-κB activation by omega-3 fatty acid conferred within the current study is viewed as a possible medical aid for inflammatory brain injury.

The histological examination of brain tissues showed degenerative changes. These changes in the brain of rats might be referred to the disturbance in oxidative, inflammatory, and deoxyribonucleic acid biomarkers. The results are in consistence with the observation of EL-Refaiy and Eissa (2013). No histopathological abnormalities were ascertained in co-administration of ω-3 FAs and Cd. Pretreatment with of ω-3 FAs followed by Cd intoxication showed return of brain tissues toward traditional morphology, however, still completely different compared with control. Omega-3 ameliorated the histological changes induced by Cd, and this directs the attention to the antioxidants as protecting measures for the neurotoxicity which was in agreement with previous results using ascorbic acid as antioxidant against Cd neurotoxicity (Afifi and Embaby 2016). Epidemiological proof suggests that a diet rich with ω-3 fatty acids promotes useful neurological and anti-inflammatory health benefits. Mounting proof suggests that these actions are mediating through each oxidative and non-oxidative route of metabolism that converts ω-3 FAs into bioactive metabolites. Interestingly, dietary supplementation with ω-3 FAs resulted in increased levels of ω-3 endocannabinoids that are formed via oxidation by cytochrome P450s. Although these metabolites exhibit the classical epoxy eicosanoid and endocannabinoid activities, their bifunctional nature imparts the ability to resolve inflammation with higher efficiency than their parent metabolites (McDougle et al. 2017). In summary, these findings represent ω-3, by virtue of its biological properties, and will function as potential therapeutic targets for diseases such as neuroinflammation and cerebrovascular disorders.

## Conclusions

In this study, the biochemical and histopathological results showed high risk of Cd neurotoxicity. Omega-3 FAs acts as antioxidant compound with neuroprotective and treatment impact versus Cd toxicity; therefore, it should be tested on alternative heavy metals and environmental toxic compounds. The histologic results confirmed the biochemical analysis. The result of the polyunsaturated fatty acid supplementation is put additional useful within the co-treatment than within the pretreatment. It is so worthy to counsel that omega-3 FAs should be included in diet to prevent/suppress the events of neurodegenerative disorders caused by continuous exposure to Cd.
